# LETTER TO THE EDITOR CONCERNING: Effectiveness of the Rigo Chêneau versus Boston-style orthoses for adolescent idiopathicscoliosis: a retrospective study, by Minsk MK, Venuti KD, Daumit GL, Sponseller PD. Scoliosis Spinal Disord. 2017 Mar 20;12:7

**DOI:** 10.1186/s13013-017-0147-6

**Published:** 2018-01-09

**Authors:** Johan L. Heemskerk, Mark Altena, Diederik H. R. Kempen

**Affiliations:** Department of orthopedic surgery, OLVG hospital, Amsterdam, The Netherlands

**Keywords:** Letter to the editor, Adolescent idiopathic scoliosis, Boston brace, Rigo Chêneau, Selection bias

## Abstract

We have read with great interest the article by Minsk et al. in Scoliosis and Spinal Disorders. However, the authors reported a conclusion that is based on possible selection bias in surgical candidates. Physicians are trained in the interpretation of scientific articles; however, not everybody is able to do this. Especially in open access journals, a biased conclusion may have big consequences and may be misleading for patients and family members who can read these articles for free on the internet.

Dear editor,

We read with great interest the article by Minsk et al. [1]. We congratulate the authors with this study and for using the recommendations of the Scoliosis Research Society (SRS) and the Society on Scoliosis Orthopedic and Rehabilitation Treatment (SOSORT) committee on bracing and non-operative Management [2, 3]. It is one of the few available studies comparing the Boston-style thoracolumbosacral orthoses (TLSO) and the Rigo Cheneau orthoses (RCO), and we encourage research in the field of conservative treatment of scoliosis. However, the conclusion of this open access article raised some confusion.

The authors conclude in the abstract that “patients treated with a RCO brace had similar baseline characteristics and brace wear time yet significant lower rate of spinal surgery”. In their conclusion, they state that “patients treated with RCOs were substantially less likely to progress to spinal surgery than those treated with Boston-style TLSOs”. Although the conclusion is supported by the significant differences between the RCO and TLSO groups, the other study results in combination with no clear description of the indication for surgery raised confusion.

Apart from the progression to surgery, the results show significant changes in major curve from baseline (6.0° versus 6.9° in the RCO and TLSO group, respectively) and percent change in major curve from baseline (18.6 versus 21.3% in the RCO and TLSO group, respectively). However, these changes do not explain the indication for surgery. Furthermore, there was no significant difference in the number of patients with curves exceeding a Cobb angle of 45° and 50° at maturity (see Table 2 in the article). In the RCO group, two patients had a major curve of 45° at skeletal maturity and even one of them had a curve bigger than 50°. None of these patients in the RCO group underwent spinal surgery. In the TLSO group, 30 patients had a major curve of 45° at skeletal maturity, while 36 patients were surgically treated or had a curve of > 45°. This suggests that six patients were surgically treated for curve magnitudes below 45°.

**Table 2:**
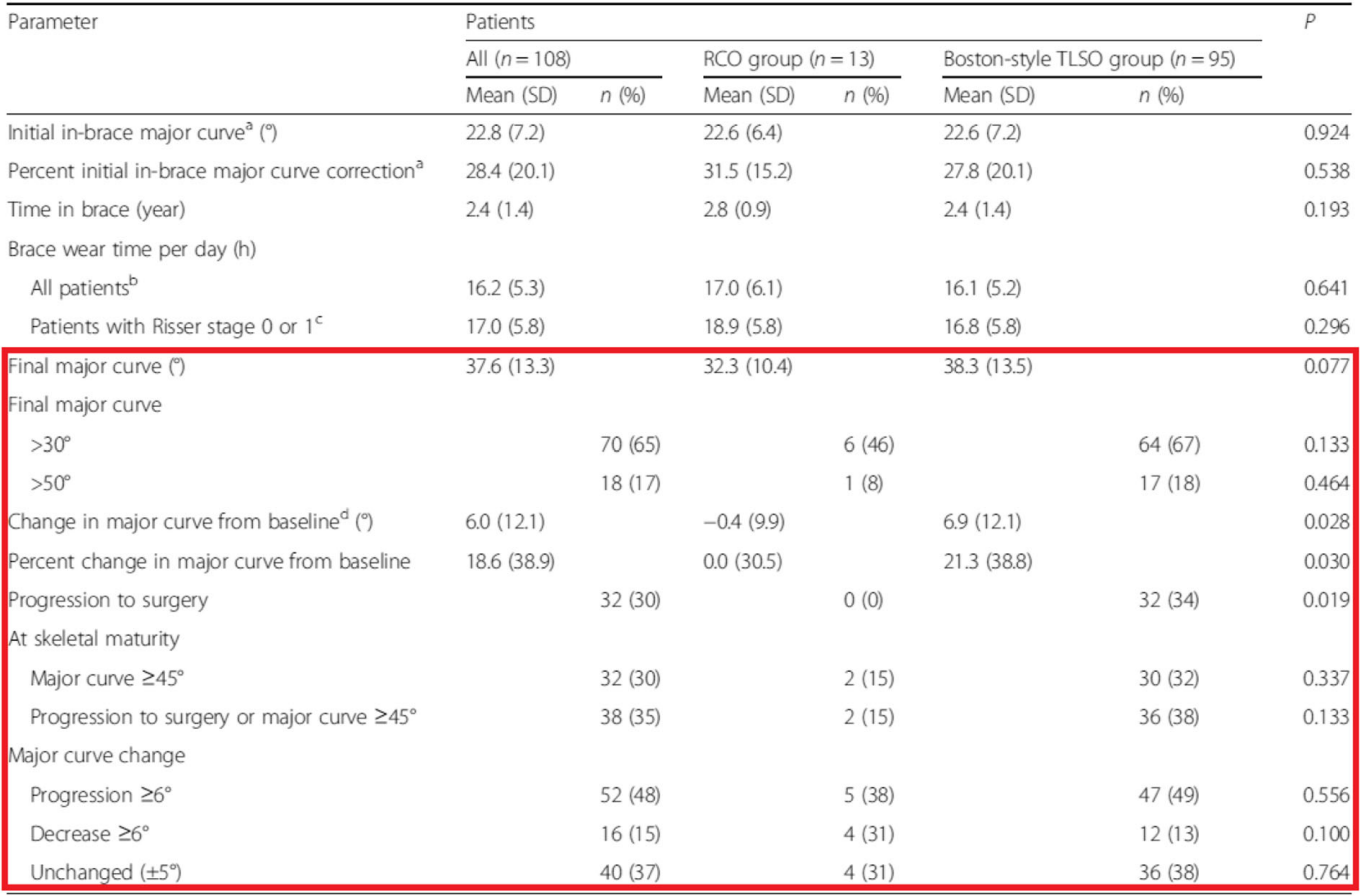
Bracing treatment and outcomes for 108 patients with adolescents idiopathic scoliosis

*SD* standard deviation, *RCO* Rigo Chêneau orthosis, *TLSO* thoracolumbosacral orthosis

^a^*n =* 83 (RCO, *n =* 12; TLSO, *n =* 70)

^b^*n =* 107 (RCO, *n =* 13; TLSO, *n =* 94)

^c^RCO, *n =* 10; TLSO, *n =* 71

^d^*n =* 95 (RCO, *n =* 11; TLSO, *n =* 84)

Although progression of the curve to a Cobb angle of 45° to 50° is a frequently reported indication for spinal surgery [4, 5], we realize that other patient characteristics may also influence treatment decisions and curves above 45° or 50° may remain stable without surgery. Apparently, indication for surgery was not only based on progression of the Cobb angle. If the indication and risk for surgical treatment differs between the groups, this may lead to bias and wrong interpretations of the results. Zaina et al. mentioned to be aware for this kind of methodological errors in scoliosis research [6]. Since there is currently no clear description of the indication for surgery, the influence of a selection bias on study results in not clear in this study.

Due to the need for long-term follow up and difficulty to measure all variables, it is difficult to design studies comparing scoliosis braces. The retrospective study by Minks et al. is one of the first good studies implementing the study recommendations of different societies. However, there is also a risk of bias from insensitivity to sample size (also known as “law of small numbers”) due to the large difference in group sizes. One case progressing to surgery in the 13 RCO patients influences the outcome and conclusions of the study. We therefore encourage the authors to continue their good work and report their results again in similar group sizes. Yours sincerely,

Johan L. Heemskerk, M.D.

Mark C. Altena, M.D.

Diederik H.R. Kempen, M.D., PhD.

## Abbreviations

RCO: Rigo Chêneau Orthosis
SOSORT: Scoliosis orthopedic and rehabilitation treatment
SRS: Scoliosis Research Society
TLSO: Thoracolumbosacral orthoses
